# Research on the Aging Characteristics of Simulated Asphalt Within Pavement Structures in Natural Environments

**DOI:** 10.3390/ma18020434

**Published:** 2025-01-17

**Authors:** Xiang Ma, Weiyi Diao, Jiachen Xu, Dongjia Wang, Yanming Hou

**Affiliations:** 1 College of Civil Engineering, Nanjing Forestry University, Nanjing 210037, China; dwi20001028@126.com (W.D.); 2097023916@njfu.edu.cn (J.X.); wangdongjia2021@163.com (D.W.); minghou412@njfu.edu.cn (Y.H.); 2 Jiangsu Province Engineering Research Center for Highway Intelligent Detection and Low Carbon Maintenance, Nanjing Forestry University, Nanjing 210037, China

**Keywords:** asphalt natural aging, pavement structure, different locations, outdoor simulation, fatigue life, cracking propagation effects

## Abstract

The global asphalt production growth rate exceeded 10% in the past decade, and over 90% of the world’s road surfaces are generated from asphalt materials. Therefore, the issue of asphalt aging has been widely researched. In this study, the aging of asphalt thin films under various natural conditions was studied to prevent the distortion of indoor simulated aging and to prevent the extraction of asphalt samples from road surfaces from impacting the aged asphalt. The aging of styrene–butadiene–styrene (SBS)-modified asphalt was simulated at four different locations on an asphalt road surface. The aging characteristics of asphalt binders across various structural layers were revealed using Fourier transform infrared spectroscopy (FTIR), atomic force microscopy (AFM), and linear amplitude scanning (LAS). The results indicate that the aging behavior of the asphalt functional group on the road surface differs from other conditions; the asphalt fatigue life of 4 months equates to the 16-month aging life of asphalt within the dense-graded asphalt road surface. After 8 months of aging, the surface smoothness of the asphalt was significantly compromised. Inside of the porous pavement, the asphalt functional group is more likely to interact with water molecules than inside the dense pavement with cracks, and the variations in roughness and the reduction in fatigue life are also more significant.

## 1. Introduction

Asphalt pavements are prone to numerous distresses due to the interplay of traffic loads and environmental factors, significantly impacting the road’s longevity and driving safety. Traffic loads have an adverse impact on pavement structure, although the raw materials of pavements are mainly affected by the natural environment. The deterioration of asphalt due to the combined influence of several environmental factors is the primary determinant of asphalt pavement aging [1,2]. During this process, the chemical composition, microstructure, and properties of asphalt change [3,4]. Therefore, understanding the fatigue damage mechanism of asphalt pavements requires in-depth research into the aging characteristics of asphalt materials under natural environmental conditions. Asphalt pavements undergo aging due to environmental variations throughout their lifespan. Pavements are completely exposed to various environmental factors, and the effects of aging are most evident. Generally, the density of pavements makes it difficult for moisture to penetrate the surface, resulting in a relatively low humidity. However, if cracks or significant gaps occur, the humidity may increase after rainfall, and drainage could become inadequate, resulting in prolonged periods of high moisture levels. At this point, aging characteristics may also differ. Therefore, enhancing the efficiency of detecting structural features of asphalt pavements and sealed cracks using innovative machine learning methods is essential [5]. Extensive studies have been conducted on the aging damage mechanism and the changes in the macro and micro properties of asphalt materials using indoor simulations, field sample extraction, and numerical simulations. From a macro perspective, the extent of asphalt aging is evaluated by comparing the differences in softening point and viscosity before and after aging [6]. The American Strategic Highway Research Program (SHRP) proposed to evaluate the rheological performance of asphalt after aging using the phase angle and complex modulus obtained from tests conducted under medium to low temperatures with a dynamic shear rheometer (DSR) [7,8]. In addition, an increasing number of precision instruments are being used to analyze the microscopic properties of aged asphalt. Many scholars use Fourier transform infrared spectroscopy (FTIR) to detect the changes in the functional groups of asphalt before and after aging [9,10,11] and thin-layer chromatography with a flame ionization detector (TLC-FID) to detect the saturates, aromatics, resins, and asphaltenes (SARA) in asphalt [12]. For microscopic characterization, fluorescence microscopes (FM), scanning electron microscopes (SEM), and atomic force microscopes (AFM) are also widely used [13,14,15,16]. AFM evaluates the mechanical properties of asphalt at a microscopic level, and scholars have highlighted their extensive application potential in conjunction with optical tweezers inside soft matter systems [17]. Hirato et al. [18] found that prolonged indoor aging increases the complex shear modulus and creep stiffness of all asphalt binders. Asphalt gradually hardens, and its creep compliance decreases, indicating a reduced ability to relax internal stresses. Li et al. [19] constructed an environmental chamber that used indoor accelerated aging to simulate the effects of high temperature, ultraviolet light, and acid rain on asphalt. In the low-temperature performance test, the acid rain solution only significantly affected the aging mid-stage. Song et al. [20] exposed asphalt to natural conditions for a year and used the aged samples to propose a quantitative evaluation index of the natural aging contribution rate (CIi) based on rheological parameters. They found that the thermal oxygen effect contributes the most to the viscoelastic properties, high-temperature rutting resistance, and low-temperature cracking resistance of SBS-modified asphalt. Wu et al. [21] indicated via FTIR experiments that the aging indicators of the on-site pavement asphalt exceed those of the base asphalt and that traffic loads accelerate the degradation of C=C double bonds, resulting in increased oxidative aging of the asphalt. Yao et al. [22] conducted a detailed analysis of the functional groups at a wavelength of 1700 cm^−1^ during the asphalt aging process using FTIR; carboxylates and ketones are the main components of the asphalt aging process. The aging-induced functional groups significantly impact high-temperature pavement distresses, including the asphalt mixtures’ rutting and fatigue performance. Wang et al. [23] found that after long-term aging, the surface morphology of the control asphalt became rough, indicating a significant increase in the heterogeneity between molecules. Aging improves the differences in properties at the interface between the honeycomb structure phase and the continuous phase, and the fatigue performance of asphalt significantly decreases after aging.

Based on the above, the current studies did not consider the differences in aging environments associated with pavement structures, resulting in a lack of targeted research. In this study, several representations of natural conditions were proposed to characterize asphalt aging in different locations; the aging properties of asphalt were analyzed under natural conditions using FTIR, AFM, and DSR, and the fatigue life was predicted using an equation.

## 2. Materials and Methods

### 2.1. Asphalt Materials

An SBS-modified PG76-22 binder (China Zhenjiang Jiangsu Yangtze River Highway Asphalt Co., Ltd., Zhenjiang, China) was employed in this study; basic properties were tested according to the methods specified in “Test Methods for Asphalt and Asphalt Mixtures in Highway Engineering” (JTG E20-2011) [24]. Test results are shown in Table 1.

### 2.2. Preparation of Asphalt Samples Under Different Aging Conditions

**Figure 1:**
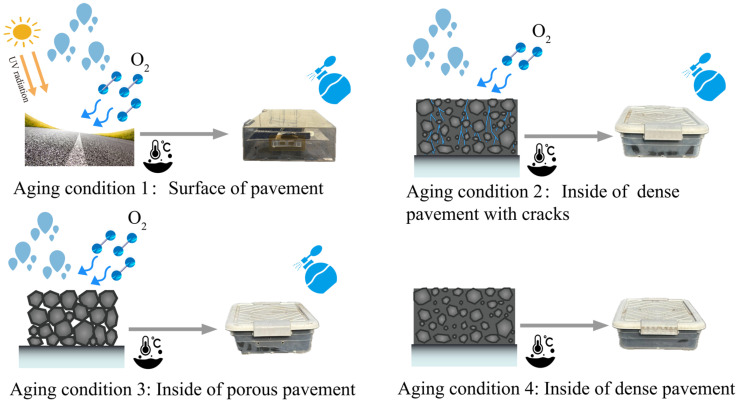
Aging diagrams of four different pavement structures.

The extraction of asphalt binder from asphalt concrete using a rotary evaporator results in different degrees of aging, leading to inaccuracies in the analysis of asphalt binder aging properties. This study designed four distinct aging conditions to more accurately represent the many natural environments encountered by asphalt pavement structures, as shown in Figure 1.

This study employed a paint applicator to create 1 mm-thick asphalt film samples of SBS-modified asphalt binder on a silicon substrate. Different environmental chambers were designed, and a fixed plate was set in the middle. After laying the samples flat on the fixed plate to ensure they did not touch each other, the environmental chambers were placed outdoors on an open roof. A watering can was used regularly to simulate rainfall, ensuring the water covered the asphalt film completely. The environment chambers were constructed as closed containers to prevent dust contamination of the samples during the natural aging process, which could affect the results of subsequent microscopic tests. The specific design of the various natural conditions was as follows:

Aging condition 1: Criteria for the surface of the pavement. The environmental chamber was constructed from high-transmittance acrylic, exhibiting a transmittance of 90% or more to ensure the efficacy of ultraviolet light. Rain was simulated via regular water sprinkling; the top of the environmental chamber was not fully sealed, and a total of six holes were opened on both sides to ensure adequate exchange of air and humidity between the chamber and the outside.

Aging condition 2: Criteria for the inside of dense pavement with cracks. A UV-resistant environmental chamber was constructed to prevent the effects of ultraviolet light and simulated rainfall through regular water spraying; the top of the environmental chamber was completely sealed, with a total of six holes opened on both sides for air and humidity exchange between the chamber and the outside.

Aging condition 3: Criteria for the inside of porous pavement. An environment chamber with UV protection was constructed to prevent the effects of UV light; water was regularly sprayed to simulate rain; the top of the environmental chamber was completely sealed, with a total of twelve holes opened on both sides for air and humidity exchange between the chamber and the outside.

Aging condition 4: Criteria for the inside of dense pavement. An environment chamber resistant to ultraviolet rays was constructed to prevent the effects of ultraviolet light; the top of the chamber was completely sealed, and no water was sprayed during the aging process. Only two holes were opened on the left and right sides of the chamber to ensure oxygen circulation.

### 2.3. Selection of Testing Frequency

The duration of this study spanned from October 2022 to February 2024, totaling 16 months, with samples collected every 4 months for testing. The study took place in Nanjing City, Jiangsu Province, China. Table 2 presents the statistical results of the environmental parameters at each aging stage in this location. The code name used in the analysis below denotes the condition number–aging duration; for example, 1–4 signifies the sample under condition 1 following an aging period of 4 months.

### 2.4. Test Methods

#### 2.4.1. Fourier Transform Infrared Spectroscopy Test

Fourier transform infrared spectroscopy (FTIR) is a qualitative and quantitative analysis method that is used to study the absorption characteristics of molecular substances in response to infrared radiation. Since various functional groups of the molecules that make up the substance have specific characteristic infrared peaks, in this study, we used the VERTEX 80 V infrared spectrometer produced by Bruker (Billerica, MA, USA) with a resolution of 4 cm^−1^, a scan count of 32, and a wavenumber range of 4000 cm^−1^ to 400 cm^−1^. During sampling, different asphalt sample films were inverted to cover the sample measurement port completely, and then the silicone plate was removed to avoid excessive heating of the samples, thus ensuring accurate test results.

#### 2.4.2. Atomic Force Microscopy Test

This study used the PF-QNM mode of atomic force microscopy by Bruker (AFM) to study the changes in surface microtopography of asphalt before and after aging under different conditions. The PF-QNM mode controls the force applied by the probe by reducing the contact area and deformation depth between the probe and the sample. The scanning size was 20 μm × 20 μm. The scanning parameter frequency was set to 0.5 Hz. The sample was scanned at a rate of 1 Hz, and the number of scan lines was 512. The roughness function in NanoScope Analysis software (v1.5) was used to calculate the average roughness (R_a_) and the root mean square roughness (R_q_) before and after asphalt aging; the equations are shown below.

(1) $$R_{q}=\sqrt{\frac{\sum {\left(Z_{i}\right)}^{2}}{N}},$$

(2) $$R_{a}=\frac{1}{N}\sum_{j=1}^{N}Z_{i},$$

where N is the number of data points selected in the chosen range and $Z_{i}$ is the Z value of a point on the surface relative to the operating state of the microscope, which can be negative.

#### 2.4.3. Linear Amplitude Sweep Test

Linear amplitude sweep (LAS) testing is an accelerated fatigue testing method primarily aimed at asphalt binder, utilizing the DSR by Bohlin (Salisbury, UK) to evaluate the fatigue performance of asphalt materials under medium temperature conditions, with a rotor diameter of 8 mm and a parallel plate gap set to 2 mm. The test temperature was set at 25 °C. The core of the LAS test is to determine the fatigue failure criterion. The analysis of loss characteristic relationships and fatigue life prediction using the linear amplitude scanning (LAS) test (AASHTO TP 101-14) [25] is derived from the viscoelastic continuous damage (VECD) model. According to Schapery’s principle of work potential [26], the internal state variable (S) is introduced into the VECD model to quantify the loss, and the equation for this value is as follows:

(3) $$\frac{\mathrm{ds}}{\mathrm{dt}}={\left(-\frac{\partial W}{\partial S}\right)}^{\alpha },$$

where W is the strain energy, S is a variable parameter characterizing the internal losses in the material, and α = 1 + 1/m.

The VECD model is capable of predicting fatigue life at different strain levels, and a power law model is usually used to fit the loss characteristic curve for fatigue life prediction, as demonstrated in Equation (4),

(4) $$\left|G^{*}\right|\sin\;\delta =C_{0}-C_{1}S^{C_{2}},$$

where $C_{0}$, $C_{2}$, and $C_{1}$ are the fitted parameters.

The relationship between fatigue life and strain is obtained by integrating the loading into the final material destruction process, as shown in Equation (5),

(5) $$N_{f}=\frac{f{\left(S_{f}\right)}^{k}}{k{\left(C_{1}C_{2}\right)}^{\alpha }}\cdot {\gamma_{0}}^{-2\alpha },$$

where $S_{f}$ is the value of material loss to the point of destruction, f is the loading frequency, and $k=1+\alpha -\alpha C_{2}$.

## 3. Results

### 3.1. Chemical Composition

With the increase in aging duration, the absorption peaks located at 966 cm^−1^ gradually decrease and disappear under all conditions, while the characteristic absorption peaks at 1031 cm^−1^ and 1700 cm^−1^ gradually increase. This is due to the increase in the degree of oxidation of the asphalt binder surface with the prolonged aging time, with the degradation of the SBS modifier causing the double bonds in polybutadiene to break. The networked cross-linked structure is destroyed, while the content of oxygen-containing functional groups gradually increases. At later stages of aging, the matrix phase of modified asphalt was mainly dominant, which led to the SBS-modified asphalt gradually becoming hard and brittle, with decreased fatigue resistance.

**Figure 2:**
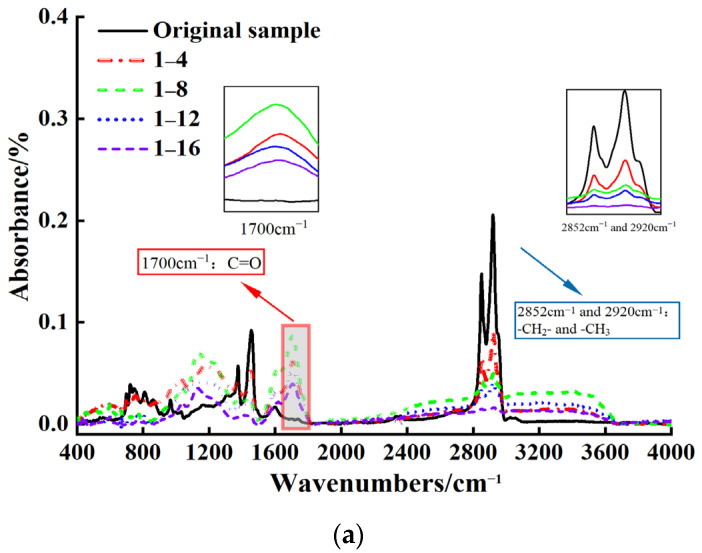

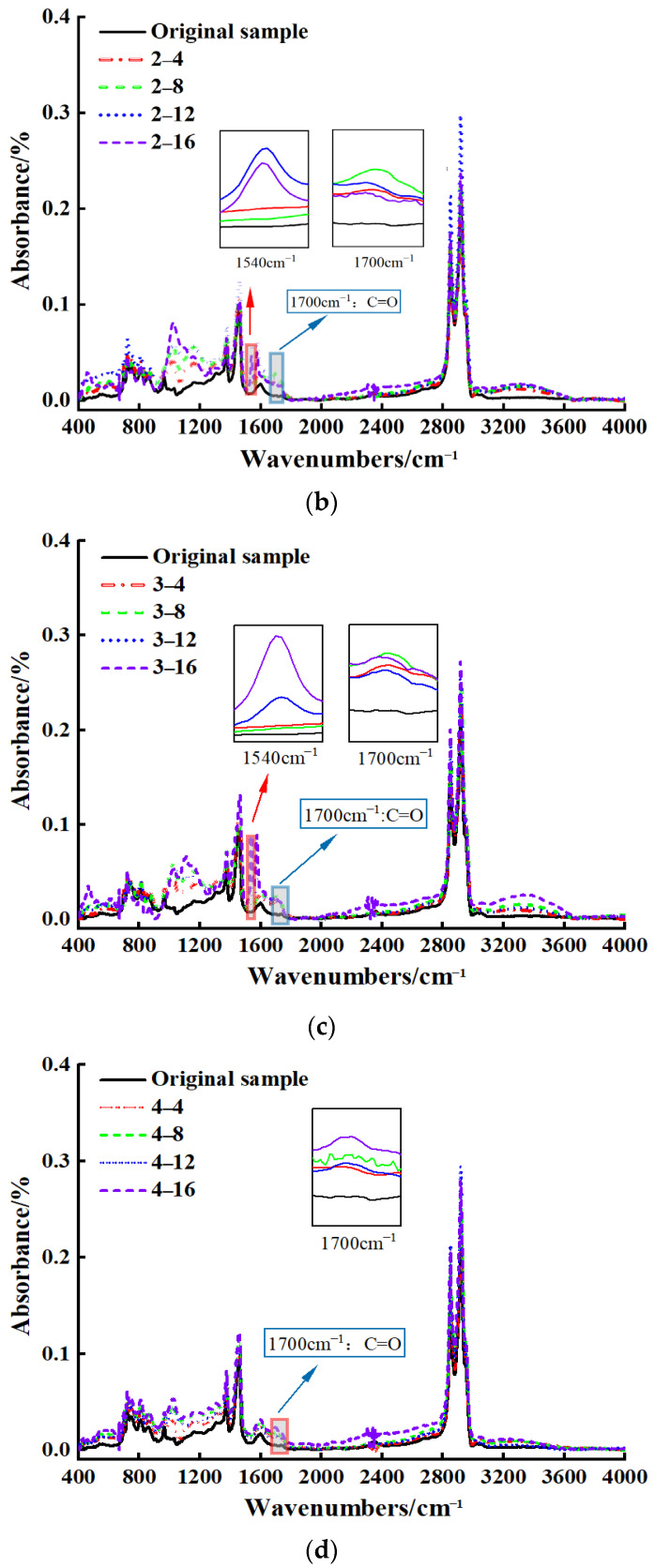
Infrared spectral wavenumber and absorption intensity diagram of SBS-modified asphalt binder under different aging conditions. (**a**) Aging condition 1; (**b**) Aging condition 2; (**c**) Aging condition 3; (**d**) Aging condition 4.

It is clearly evident from Figure 2a that the carbonyl absorption peak’s intensity is the greatest on the pavement surface compared to the other three conditions. This is due to ultraviolet radiation; the single bond C-O in asphalt binder absorbs ultraviolet light, transitioning from the ground to the excited state and generating active free radicals. These free radicals are highly active and easily react with oxygen to form C=O [27]. The radiation value in this study peaked within 8 months, and an absorption peak due to carbonyl compounds was also clearly visible during this period. In addition, the absorbance intensity of the methyl (2920 cm^−1^) and methylene peaks (2852 cm^−1^) of the asphalt binder in aging condition 1 significantly decreased with the increase in aging time. This is due to the continuous breaking of C-C/C-H bonds in asphalt binder, and when combined with molecular oxygen in a humid and high-temperature environment, they transform into C-O-C/C-O-H bonds [28], leading to rapid oxidation and aging reactions. This chemical change led to the breaking or oxidation of the carbon–hydrogen bonds in CH_2_ and CH_3_ groups, weakening the intermolecular bonding forces of the asphalt binder. The change in the molecular structure of the asphalt binder causes more cracks to form on its surface, leading to increased susceptibility to fatigue damage under cyclical loading.

From Figure 2b,c, it can be concluded that compared to dense pavement with cracking, porous pavement is characterized by a greater fluctuation in the intensity of the absorption peak at 1600 cm^−1^ on the left side. This fluctuation is closely related to the increase in humidity over the 12-month aging period. In high-humidity environments, the moisture content in the asphalt binder increases, and water molecules interact with its functional groups, leading to changes in the molecular structure [29,30]. At the same time, the presence of water molecules weakens the intermolecular interactions between asphalt molecules, establishing cohesion, which makes the asphalt binder more susceptible to fatigue damage when subjected to repeated loading [31].

### 3.2. Microstructure

#### 3.2.1. Surface Microtopography Map

Research [32,33] indicates that the main component of the protruding parts of the honeycomb structure is asphaltene, while the subsiding areas consist of saturated phenols. The white protrusions of the peak-shaped structure, the yellow protrusions below, and the yellow protruding part at the tail are composed of resins. Aromatic phenols adsorb around the asphaltene and resin, forming relatively horizontal parts in the microscopic structural diagram of asphalt. Under aging condition 1, after 8 months of aging, the surface flatness of the asphalt binder samples did not meet the requirements of atomic force microscopy testing, and they also failed to reach a fluid state under high-temperature heating, making it impossible to observe their morphology using atomic force microscopy. This is because prolonged ultraviolet aging resulted in severe cracking on the surface of the asphalt binder. The asphalt binder samples on the pavement were only tested after 8 months of aging.

**Figure 3:**
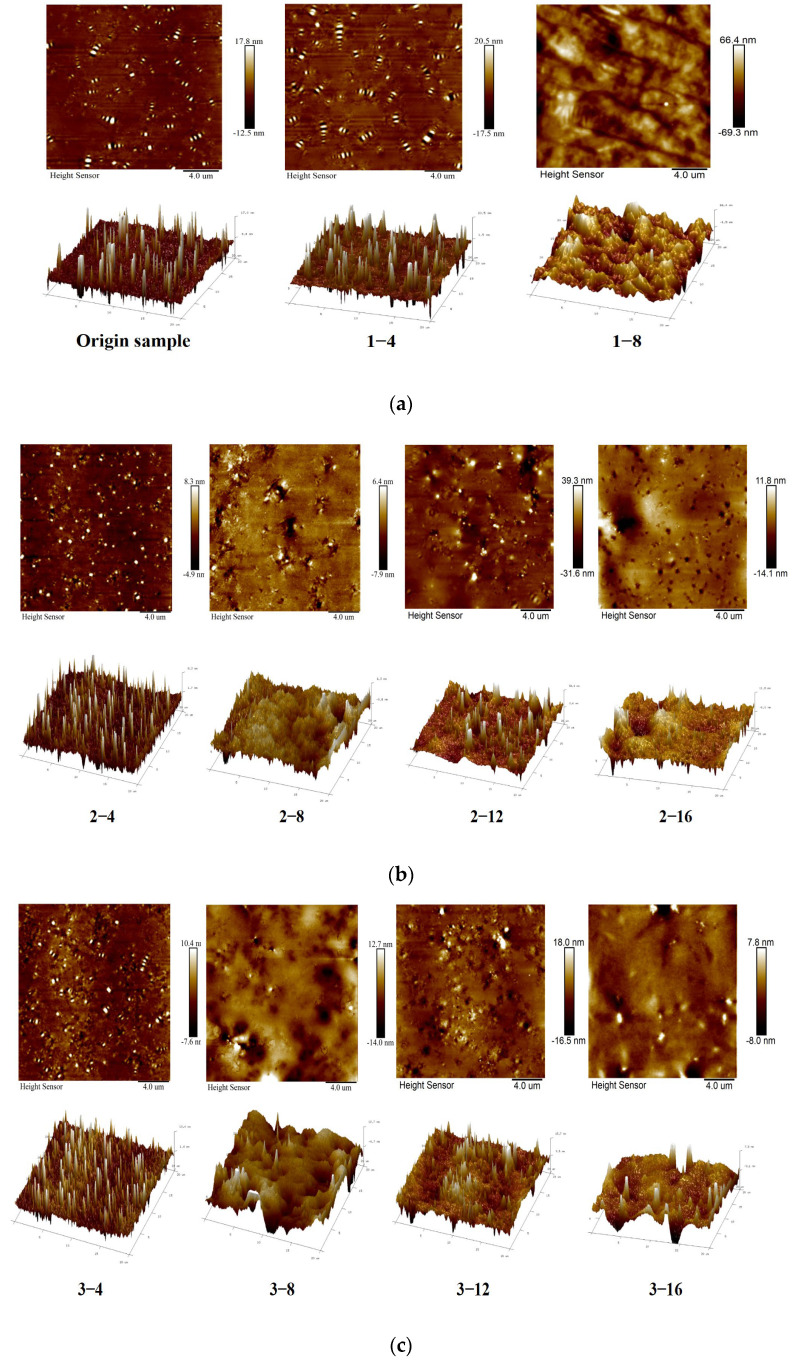

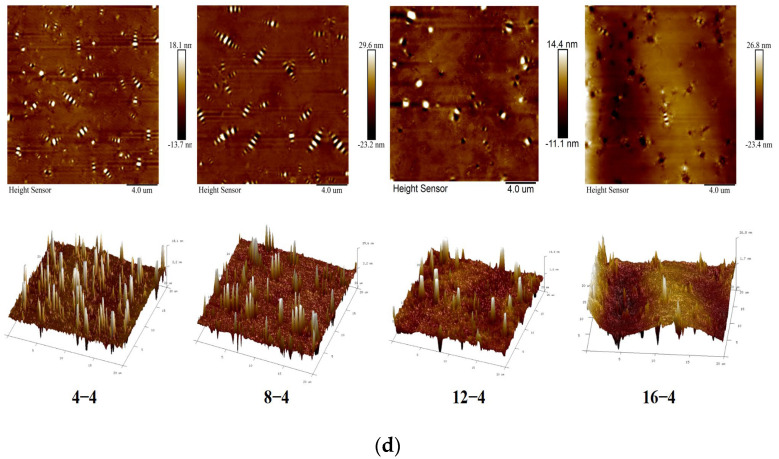
Microscopic morphology of SBS-modified asphalt binder before and after aging under various aging conditions. (**a**) Aging condition 1; (**b**) Aging condition 2; (**c**) Aging condition 3; (**d**) Aging condition 4.

The micro-morphology of the SBS-modified asphalt binder before and after aging under various working conditions is shown in Figure 3. The surface micro-morphology images of pavement and the inside of dense pavement show that when the duration of asphalt binder aging increases, the number of honeycomb structures decreases correspondingly. In addition, some honeycomb structures may merge and coalesce during the aging process, resulting in these structures being distributed more densely on the surface of the asphalt binder. This is because non-polar components in asphalt binder (saturated and aromatic phenols) gradually convert into polar components (resins and asphaltenes), causing the asphalt binder to adopt a gel-like structure. The molecular mobility of asphaltenes decreases, leading to their small honeycomb structures aggregating into larger honeycomb structures. It can be clearly seen from the 3D diagram that the white protruding parts of the honeycomb structure represent the glue, and the yellow protruding parts below and at the tail become more prominent as the polar component increases. As the SBS-modified asphalt binder becomes harder and more brittle, its resistance to fatigue failure decreases.

The honeycomb structure in porous and cracked, dense pavements significantly changed compared to the two conditions. The figure shows that in porous and dense, cracked pavements, the honeycomb structure on the asphalt binder surface became more prominent but decreased in size after four months of aging. In the asphalt binder sample from inside the cracked pavement, honeycomb valley structures aggregated, with some areas gradually transforming into honeycomb peak structures. This indicates that as aging progresses, water gradually penetrates the asphalt binder, interacting with its components. This destroys the colloidal structure in the dry state and leads to microstructural breakdown and delamination, forming a certain number of protrusions. At the same time, water can penetrate the asphalt binder surface film, creating capillary channels formed by micro-bubble activation centers, further exacerbating the damage to the microstructure of SBS-modified asphalt binder [34]. As the aging time increases, the area of the honeycomb structure on the asphalt binder surface gradually decreases. This phenomenon is due to the prolonged contact between water and the asphalt binder, leading to the emulsification of the asphalt binder and thus reducing its adhesion and cohesion, which in turn weakens its ability to resist fatigue damage [35].

**Figure 4:**
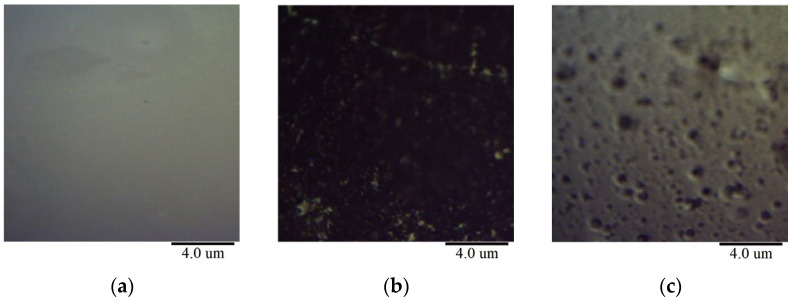
Morphological changes in different aging states under atomic force microscopy. (**a**) Origin sample; (**b**) Ultraviolet aging; (**c**) Hygro-oxidative aging.

To observe the surface changes more thoroughly, the atomic force microscope’s camera function was used to capture the asphalt binder surface before probe contact, as shown in Figure 4. It is evident that the damage patterns of the asphalt binder surface with and without ultraviolet light are drastically different. In aging condition 1, the surface was severely damaged due to the influence of ultraviolet light: it had almost no smooth areas and exhibited black, particle-like damage. At the same time, lighter components were transformed into heavier components, suggesting that these black particles may be the colloidal and asphaltene materials within the asphalt. In contrast, under other conditions without direct sunlight, a surface with noticeable pits was observed. This may be due to the dissolution of surface molecules or other physical processes.

**Figure 5:**
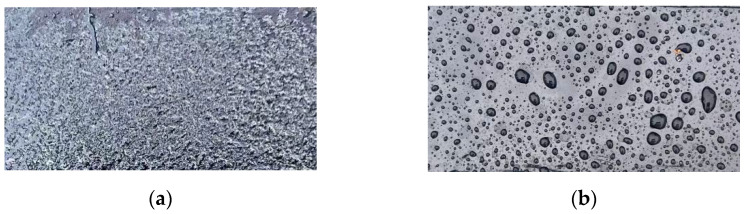
Water distribution pattern after watering under aging with different environmental factors. (**a**) Ultraviolet effects; (**b**) No ultraviolet effects.

Similar phenomena were also observed during sample preparation, as shown in Figure 5. After 12 months of aging, the surface of the pavement surface asphalt binder that was in full contact with the natural environment was seriously damaged, leading to easy water penetration and coverage. Due to the short aging cycle and lack of drainage control, water is distributed as droplets on the asphalt mix’s surface inside dense pavements. Because of the surface’s potholes and depressions, water accumulation points are formed. Moreover, the protective film on the surface of the asphalt material has not been completely destroyed, so when water is poured on the asphalt surface, water droplets are distributed on the surface of the asphalt binder, indicating hydrophobicity.

#### 3.2.2. Surface Roughness

In order to better quantitatively analyze the surface morphological changes in the SBS-modified asphalt binder’s surface after aging, the roughness value was calculated according to the formula; the calculation results are shown in Table 3. The surface roughness of the road surface asphalt binder increased gradually with the increase in aging time because, under these conditions, the SBS-modified asphalt binder was exposed to air. Oxygen reacted with the surface of the asphalt binder, causing it to harden and become brittle, and the surface roughness also gradually increased. The difference between Ra and Rq gradually decreased due to the adjustment of the microstructures of the SBS-modified asphalt binder surface under ultraviolet aging. During the aging process, some substances entered the microscopic defects on the surface, filling the depressions on the surface and thereby reducing surface irregularities.

The effect of humidity on the surface roughness of the SBS-modified asphalt binder under the internal environment of porous pavement and dense pavement with cracking has produced a series of impacts. During the four-month aging period, the polymer phase in the SBS-modified asphalt binder degraded significantly, reducing the differences between its phases. At this time, in a low-temperature environment, water vapor formed a “protective film” on the surface of the asphalt binder, making the surface of the asphalt binder sample relatively smooth and thereby decreasing the roughness. As summer temperatures and aging durations gradually increased, the strong oxidizing effect of water vapor led to a significant loss of non-polar components within the asphalt binder, subsequently affecting the properties of the asphalt binder surface. Meanwhile, at high temperatures (summer), the evaporation rate of moisture on the asphalt binder surface accelerated, and oxidation reactions of the asphalt binder were promoted, resulting in severe damage to its surface. This process is reflected in the 3D graphs shown in Figure 3b,c, where numerous peaks and valleys appear on the asphalt binder’s surface, leading to a gradual increase in R_q_ − R_a_.

The roughness of the asphalt binder tends to increase initially and then decrease inside the dense pavement environment due to the fact that certain components of the asphalt binder may undergo decomposition, polymerization, or other chemical reactions in the absence of oxygen. As the aging process progresses, some volatile components in asphalt gradually dissipate. At the same time, some aging by-products or surface polymers form, filling in the original depressions, thereby reducing the roughness of the asphalt binder surface.

### 3.3. Fatigue Behavior

#### 3.3.1. Loss Characteristic Curves

**Figure 6:**
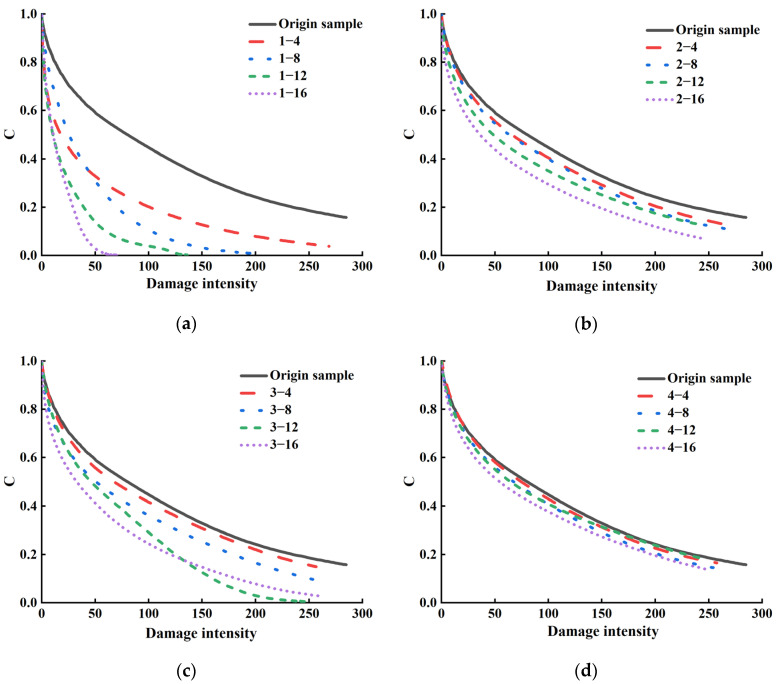
SBS-modified asphalt binder damage intensity and material integrity coefficient C value curves under different aging conditions. (**a**) Aging condition 1; (**b**) Aging condition 2; (**c**) Aging condition 3; (**d**) Aging condition 4.

Figure 6 shows the loss characteristic curves of the SBS-modified asphalt binder before and after aging under different conditions. By fitting the loss characteristic curve, the material integrity of SBS-modified asphalt binders at different loss intensities can be determined. At a given loss intensity, asphalt samples with higher integrity factor C values will exhibit better material integrity and fatigue resistance because they exhibit better durability under cyclic loading. It can be seen from Figure 6 that with the increase in aging days, the damage characteristic curves of the SBS-modified asphalt binder show a downward trend under all working conditions, and the loss intensity and material integrity of the SBS-modified asphalt binder gradually decrease. As the aging degree increases, the fatigue resistance and durability of the SBS-modified asphalt binder under cyclic loading will decrease accordingly. The most evident feature of the damage curve of the asphalt binder on the surface of pavements is the downward shift, which indicates that ultraviolet ray radiation will accelerate the aging, destroy the internal molecular chains, and, thus, reduce the fatigue resistance and durability under cyclic loading of the SBS-modified asphalt binder.

#### 3.3.2. Shear Stress–Strain Curve

**Figure 7:**
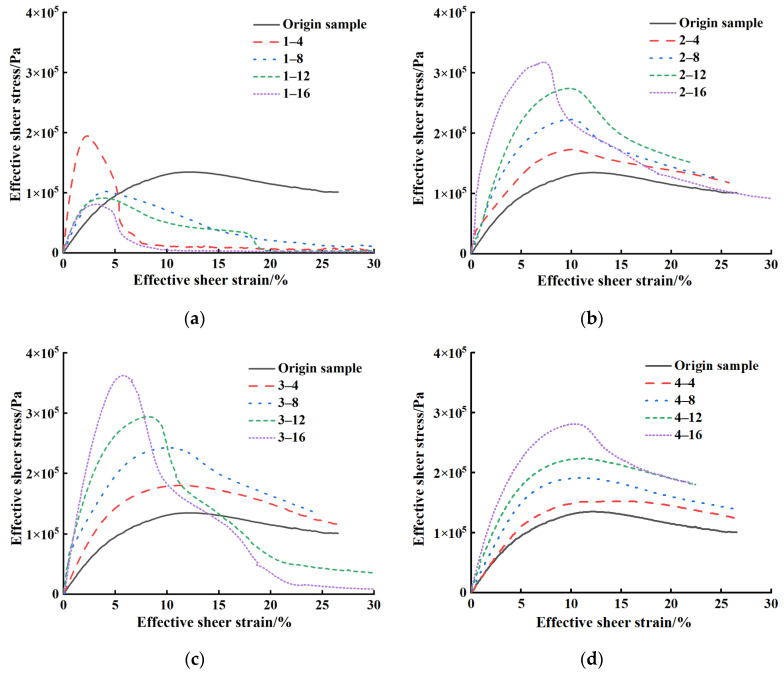
Shear stress–strain curves of SBS-modified asphalt binder before and after aging under different working conditions. (**a**) Aging condition 1; (**b**) Aging condition 2; (**c**) Aging condition 3; (**d**) Aging condition 4.

Figure 7 shows the SBS-modified asphalt binder’s shear stress–strain curves before and after aging under different working conditions. In the initial stage, the shear stress of the SBS-modified asphalt binder increases linearly with the increase in shear strain, indicating that the asphalt binder has not experienced any loss during this phase. However, as the strain increases, the shear stress growth rate gradually slows until the SBS-modified asphalt binder reaches its peak shear stress under shear strain. At this point, the shear stress of the SBS-modified asphalt binder begins to decrease as the shear strain increases, as the loss of the SBS-modified asphalt binder occurs at this stage [36].

**Figure 8:**
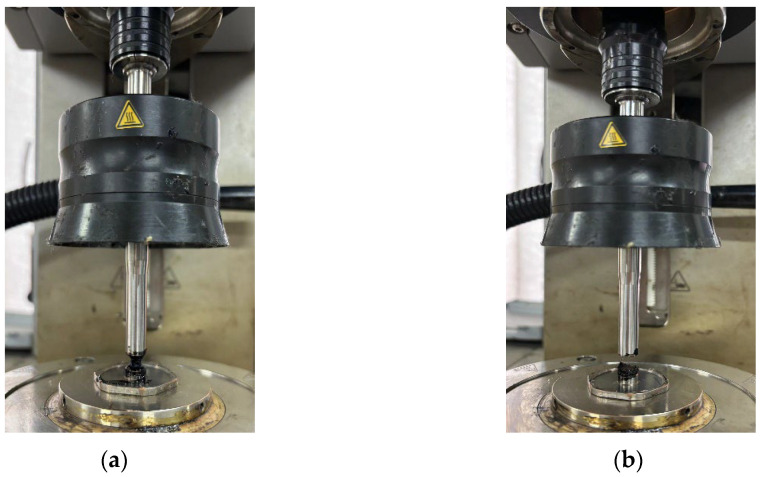
Graph of LAS test results. (**a**) Normal damage pull-off; (**b**) Abnormal damage pull-off.

The performance changes in the SBS-modified asphalt binder are reflected in the increase in peak stress and the overall shape and characteristics of its stress–strain curve. In the early stages of aging, the peak stress of asphalt may slightly increase, but the overall shape of the curve does not significantly change, which may indicate that the molecular structure of the asphalt binder has not yet been noticeably affected. As aging time progresses, the molecular chains of the asphalt binder may gradually break, cross-link, or undergo other chemical changes, resulting in significant alterations to its overall performance. The change will cause the stress–strain curve of the asphalt binder to show a more pronounced upward trend, and the slope before and after the peak stress will gradually increase as the asphalt binder approaches or has already reached its fatigue limit. Asphalt binders’ deformation characteristics and load-bearing capacity may be severely affected during this process, leading to decreased fatigue performance and a shortened lifespan in actual engineering. The simulated surface layer asphalt binder sample became too hard and brittle after aging for 8 months, and it could not fully adhere to the test platform during the test. Therefore, the stress–strain curve has a certain experimental error. The sample diagram is shown in Figure 8.

According to previous studies [11,37], the shear strain corresponding to the peak shear stress can be regarded as the yield strain of the asphalt binder. As the aging time increases, the yield strain of SBS-modified asphalt binder gradually decreases because, during the aging process, SBS-modified asphalt binder may undergo chemical reactions such as oxidation and polymerization, so the hardness and brittleness of the asphalt binder increase. This hardening makes it easier for asphalt to undergo brittle fracture under external forces rather than elastic deformation; thus, the yield strain gradually decreases.

The tensile strain at the surface of the pavement asphalt binder rapidly decreased to 0–5% within 4 months of aging, even exceeding the asphalt binder samples aged for 16 months under other conditions, which indicates that the action of ultraviolet radiation accelerates the hardening and brittleness of the SBS-modified asphalt binder, causing its tensile strain to rapidly decrease. Additionally, the yield strain of the SBS-modified asphalt binder in dense pavement with a cracking environment decreased by 5.6%, 10.2%, and 19.1% within 8–16 months, while the decrease in large void pavement was 5.9%, 17.9%, and 29.4% within the same period. Due to the increase in summer temperatures, the interaction rate of oxygen and humidity with the asphalt binder surface was promoted, reducing the fatigue resistance of SBS-modified asphalt binder. It indicates that the effect of temperature on the two environmental factors is oxygen > humidity. Meanwhile, as the aging process progresses, the yield strain of the SBS-modified asphalt binder also decreases gradually.

#### 3.3.3. Fatigue Life Prediction

Asphalt binders’ fatigue life (N_f_) in different aging stages can be predicted using the equation that Hintz et al. [38] proposed. Based on the pavement’s actual stress state, asphalt’s fatigue life should be studied primarily under 2.5% strain and 5.0% strain (N_f2.5%_, N_f5.0%_). Therefore, Table 4 lists the values of N_f2.5%_ and N_f5.0%_ for SBS-modified asphalt binders at different aging stages. The increase in aging duration under different working conditions causes the N_f_ value of the SBS-modified asphalt binder to decrease gradually. The fatigue life of the asphalt at the pavement surface after 4 months of aging is consistent with the fatigue life of the asphalt binder inside the dense pavement after 16 months of aging. Compared to winter, the fatigue life of the asphalt binder at the pavement’s surface decreases by 8% in summer. This is due to the synergistic effects of heat, solar radiation, and humidity in the pavement surface environment, which cause severe aging of the SBS-modified asphalt binder, significantly reducing its fatigue resistance. Fatigue failure can occur during the service life of the pavement. The fatigue life shows a significantly declining trend within 4–12 months under all four aging conditions, which is due to the fact that this period corresponds to the summer season of 2023. During this time, the temperature in Nanjing gradually rises, reaching its peak between June and October. Compared to winter, summer is the main period during which the fatigue life of asphalt decreases.

YI et al. [39] found through research that ultraviolet light causes the hardening of asphalt and an increase in the loss of complex modulus, which can severely reduce the fatigue life of asphalt binder. However, actual data show that the N_f_ of the asphalt binder in a dense pavement with a cracking environment decreased by 57% after aging for 12 months compared to 8 months, whereas in a porous pavement environment, it decreased by 67%. This indicates that although the latter drains moisture more quickly, its more thorough exposure to air in the natural environment leads to a more significant reduction in fatigue characteristics. It can be seen that high temperature combined with oxygen is also an important reason for the reduction in asphalt binder fatigue characteristics, and the increase in temperature can further promote the aging reaction of oxygen and humidity with the asphalt binder. The asphalt binder in a dense pavement environment is only influenced by thermal factors, and its surface is covered with fabric to prevent ultraviolet rays in order to avoid photodegradation. Therefore, the heat absorbed by its surface is also lower than that under other conditions, while the decline in the fatigue life of the asphalt binder in the dense road surface environment and the degree of fatigue loss during the aging process are the smallest. Additionally, due to the short aging period of 4 months, the SBS-modified asphalt binder did not show significant aging after being compacted in the pavement environment; therefore, the fatigue life measured at this stage was higher than that of the original sample due to experimental error.

## 4. Conclusions

This study investigates the performance of SBS-modified asphalt binders after aging, focusing on the microstructure, surface morphology, and macro performance. Through experimental results and a statistical analysis simulating asphalt binders under different pavement structures under natural conditions, the following conclusions can be drawn:

(1) The aging degree of the asphalt binder on the pavement’s surface is the worst, as the carbon–hydrogen bonds in the CH_2_ and CH_3_ groups of asphalt break or oxidize under the influence of natural factors, weakening the intermolecular bonding strength of the asphalt. As the aging duration increases, the surface roughness of the SBS-modified asphalt binder gradually increases, leading to severe oxidation of the asphalt binder surface, making it extremely hard and brittle.
(2) The asphalt in dense pavements with cracks will absorb moisture from the environment. These water molecules interact with the functional groups in the asphalt binder, resulting in new absorption peaks in the infrared spectrum. A honeycomb-like structure aggregation phenomenon appears on the surface, and the area of the honeycomb structure gradually decreases. The asphalt binders’ ability to resist fatigue damage is further weakened.
(3) In high-temperature environments during summer, although the asphalt binder inside the porous pavement dissipates moisture more quickly, it comes into complete contact with the air in the natural environment, resulting in a greater decline in fatigue life compared to the asphalt binder in dense pavements with cracks. The breaking and cross-linking of asphalt molecular chains cause the surface structure to become irregular and fragile, resulting in more severe damage to the microscopic morphology.
(4) For dense pavements, the variation in various aging parameters is minimal under various working conditions. Increasing the density of asphalt concrete and reducing the contact between the asphalt binder and external environments can significantly decrease the aging degree of asphalt binder.

This study has limitations, such as the insufficient aging time of the asphalt binder in natural environments, which requires longer validation, and issues such as the inability to control the drainage rate in the experimental chamber. In the future, we will continue to follow up on these findings as the aging time increases, further observing the changes in the properties of asphalt binders. However, the study period will only be extended to a certain extent, with a fixed study period of one year. In addition, we will also predict the aging condition of the asphalt binders in the pavement site through numerical simulations and other methods to advance future research in this area.

## Figures and Tables

**Table 1:** Properties of SBS-modified asphalt binder.

Technical Index		Test Result	Technical Requirement
Penetration (25 °C, g, 5 s) (0.1 mm)		55.5	40~60
Ductility (5 °C, 5 cm/min) (cm)		37.4	≥20
Softening point (°C)		78.5	≥60
After thin film oven test (TFOT)	Weight loss (%)	−0.15	±0.6
	Penetration comparison (%)	73.2	≥65
	Ductility (cm)	25	≥20
Performance grade		PG76-22	

**Table 2:** Representative values of environmental factors at each aging stage.

Aging Stage	Accumulative Aging Time	Four Months	Eight Months	Twelve Months	Sixteen Months
	Increased Aging Period	24 October 2022–24 February 2023	24 February 2023–24 June 2023	24 June 2023–24 October 2023	24 October 2023–24 February 2024
Temperature (°C)	Average value	7.6	18.5	25.5	7.1
	Maximum value	25.0	35.8	38.5	29.4
Humidity (%)	Average value	71.6	68.6	81.7	72.7
	Maximum value	100	100	100	100
Radiation (W/m^2^)	Accumulative value	334,780	5,663,308	493,785	356,724
	Average value	112.49	196.63	168.64	119.28
	Maximum value	765.9	993.4	953.1	757.0

**Table 3:** Surface roughness of SBS-modified asphalt binder before and after aging under each condition.

Sample Number	R_a_	R_q_	R_q_ − R_a_
Original sample	1.515	3.270	1.755
4-S-1	3.380	4.710	1.330
8-S-1	3.750	4.850	1.100
4-S-2	0.921	1.550	0.629
8-S-2	1.357	2.060	0.703
12-S-2	2.470	3.177	0.707
16-S-2	2.760	3.760	1.000
4-S-3	1.383	2.487	1.104
8-S-3	1.798	2.598	0.800
12-S-3	2.543	3.590	1.047
16-S-3	3.177	4.410	1.233
4-S-4	1.735	3.405	1.670
8-S-4	2.095	3.815	1.720
12-S-4	2.087	3.117	1.030
16-S-4	1.773	2.710	0.937

**Table 4:** $N_{f}$ values of SBS-modified asphalt binder under four conditions with different aging times.

Fatigue Life	$N_{f2.5\%}$				$N_{f5\%}$			
Aging condition	1	2	3	4	1	2	3	4
Original sample	31,577				4128			
Four months	15,986	22,862	21,989	32,807	2593	3690	3553	5292
Eight months	2687	19,625	18,437	22,575	435	3165	2959	3640
Twelve months	1759	8352	5956	20,378	282	1341	950	3025
Sixteen months	434	4254	2326	17,161	70	682	376	2771

## Data Availability

The original contributions presented in this study are included in the article. Further inquiries can be directed to the corresponding author.

## Abbreviations and Symbols

The following abbreviations are used in the manuscript:

PG	Performance Grade
JTG	China’s transportation industry standards of the People’s Republic of China
UV	Ultraviolet
PF-QNM	Peak Force Quantitative Nanomechanical Mode

The following symbols are used in the manuscript:

α	Material constants in the undamaged state
m	Estimation of parameters for the primary curve’s slope
δ	Phase angle
γ_0_	Shear strain at the beginning of the cycle
